# Supplementary material for: RAB1A is a novel vulnerability in uveal melanoma revealed by dual inhibition of MNK1/2 and mTOR
Source: Cell Death Differ. 2026 Feb 18;33(9):1891–906. doi: 10.1038/s41418-026-01684-y (PMC13545286; doi:10.1038/s41418-026-01684-y)

Figure 1D T143

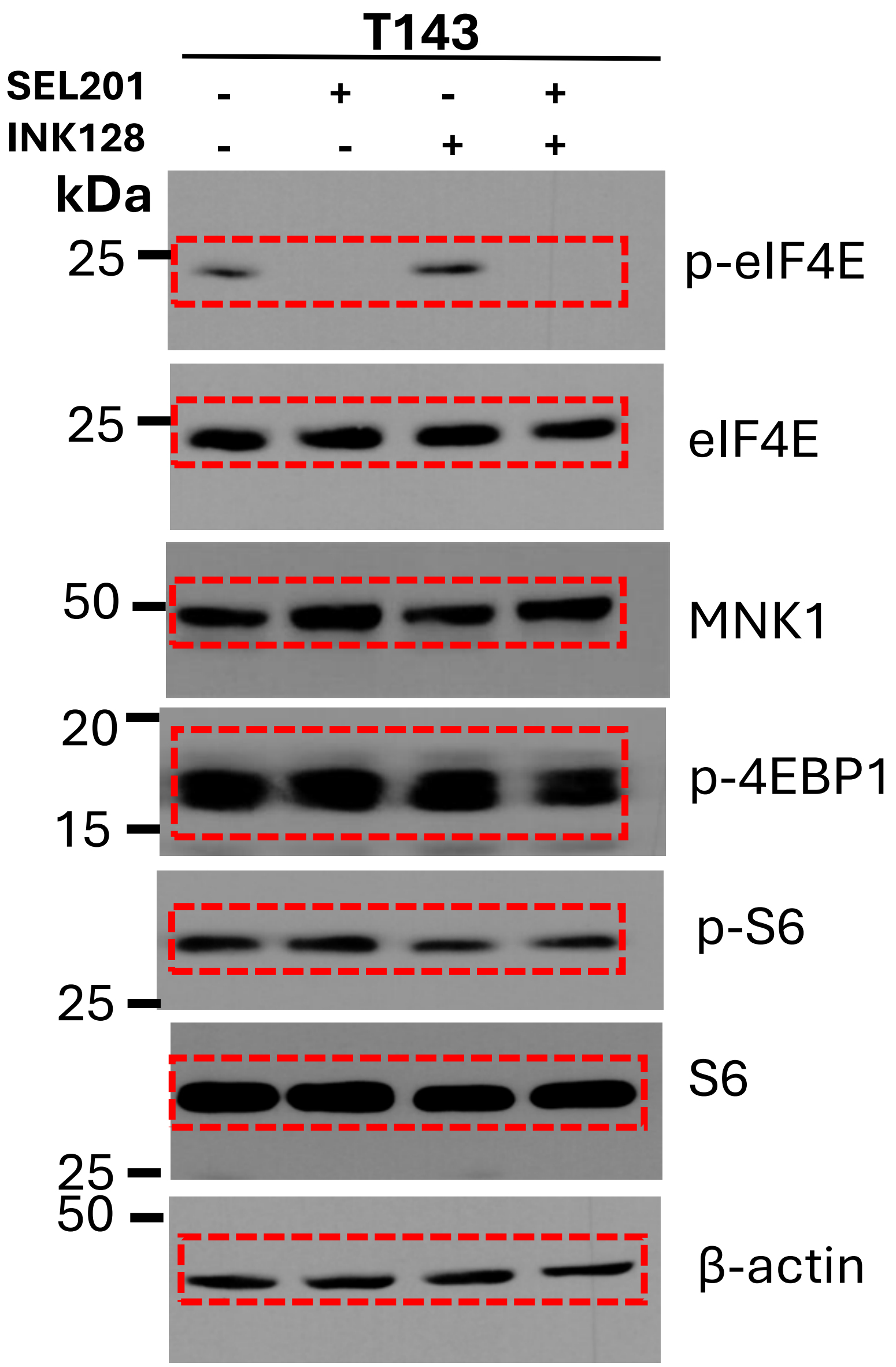

Figure 1D T128

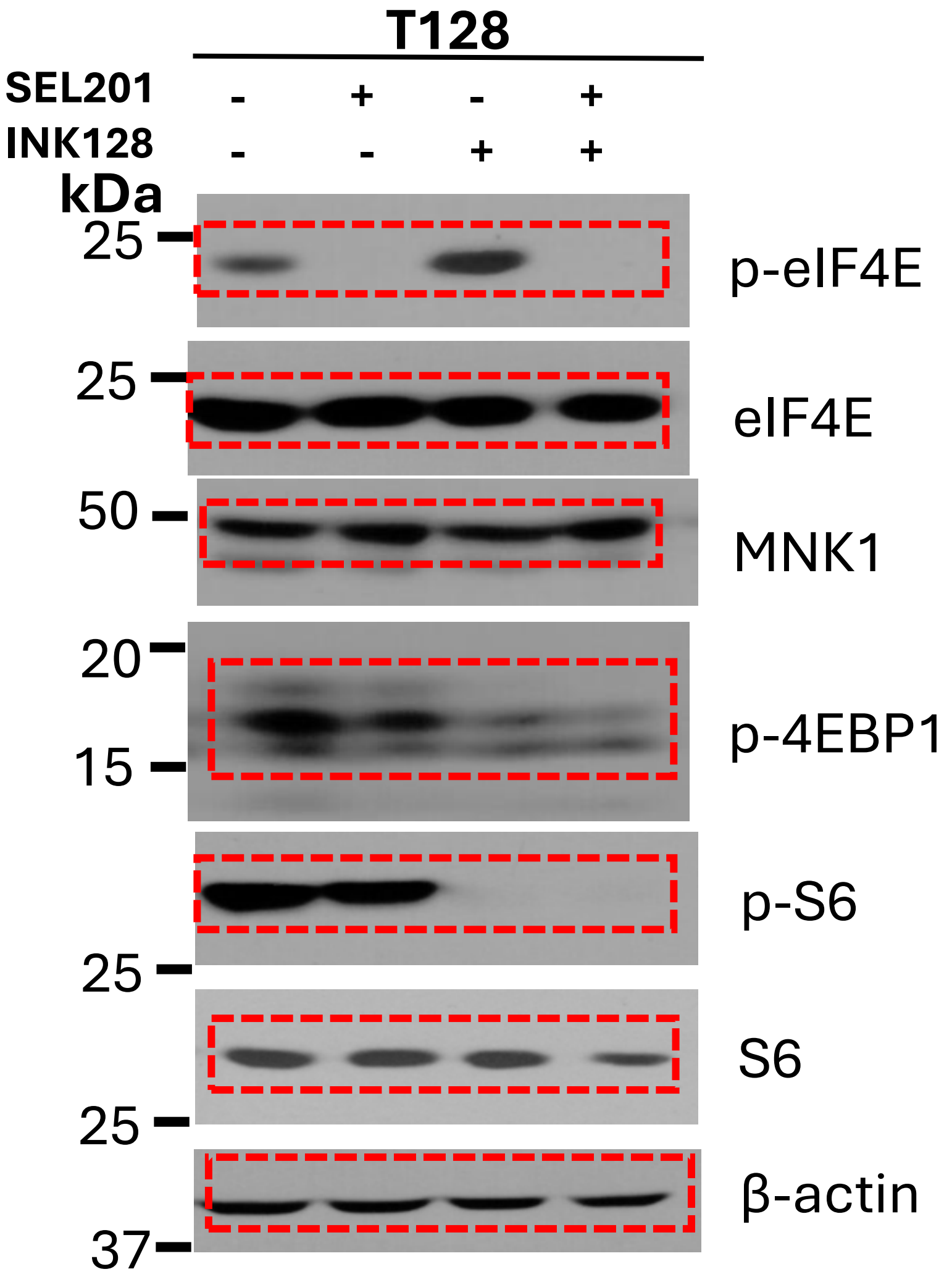

Figure 1D 92.1

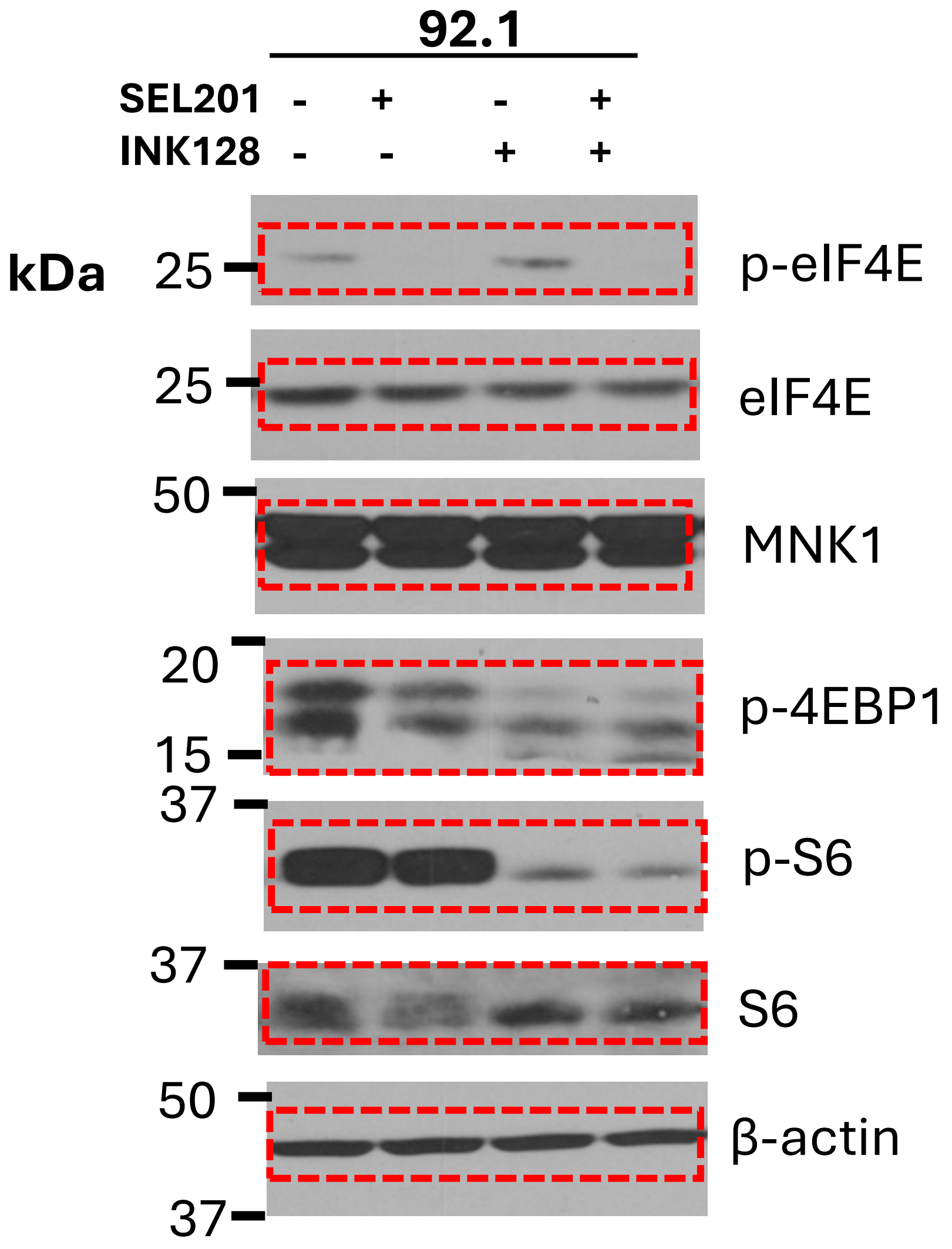

Figure 1D MEL290

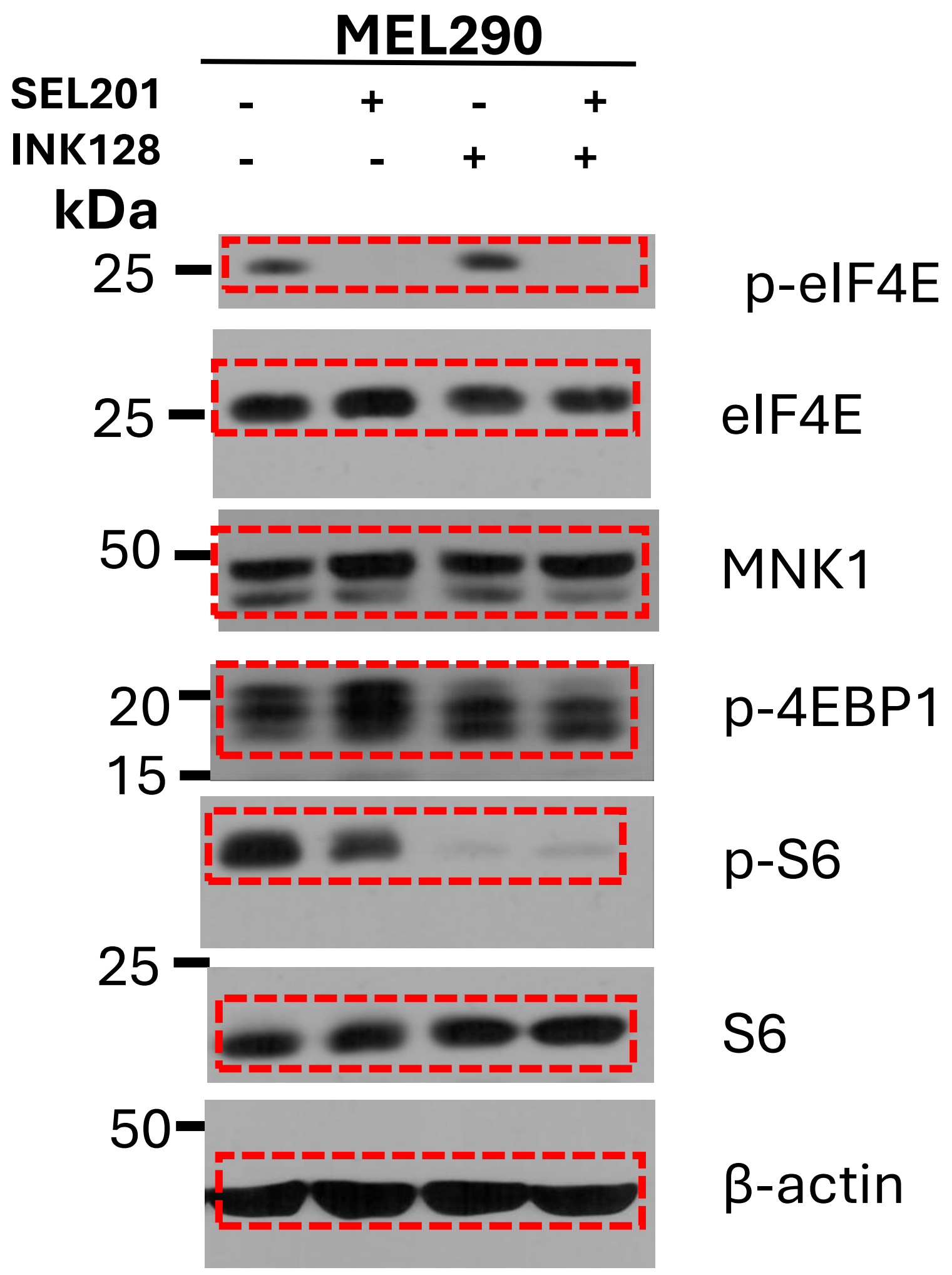

Figure 2E T128, T143

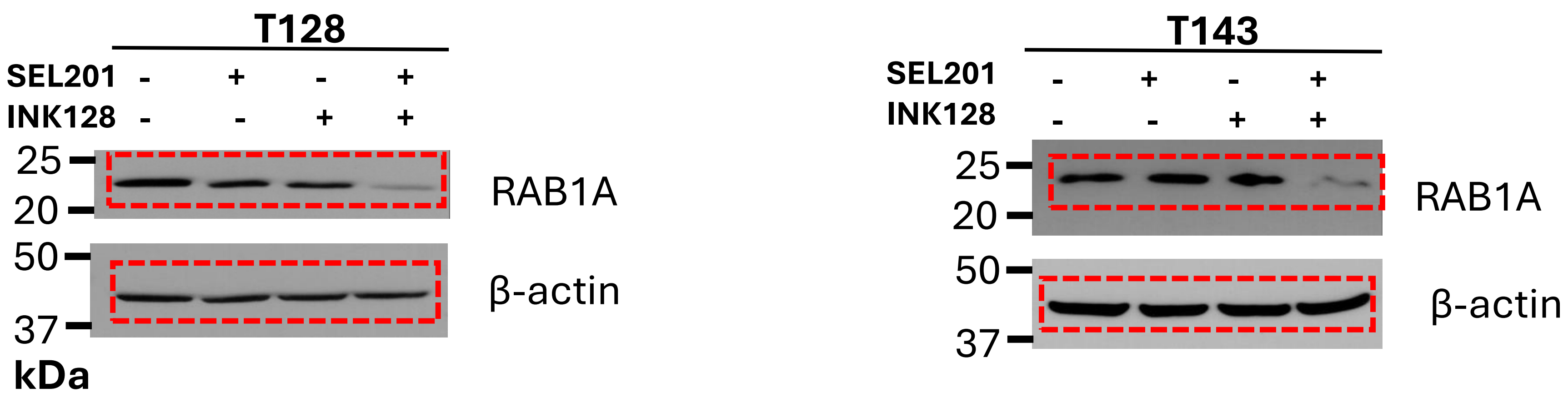

Figure 2E 92.1, MEL290

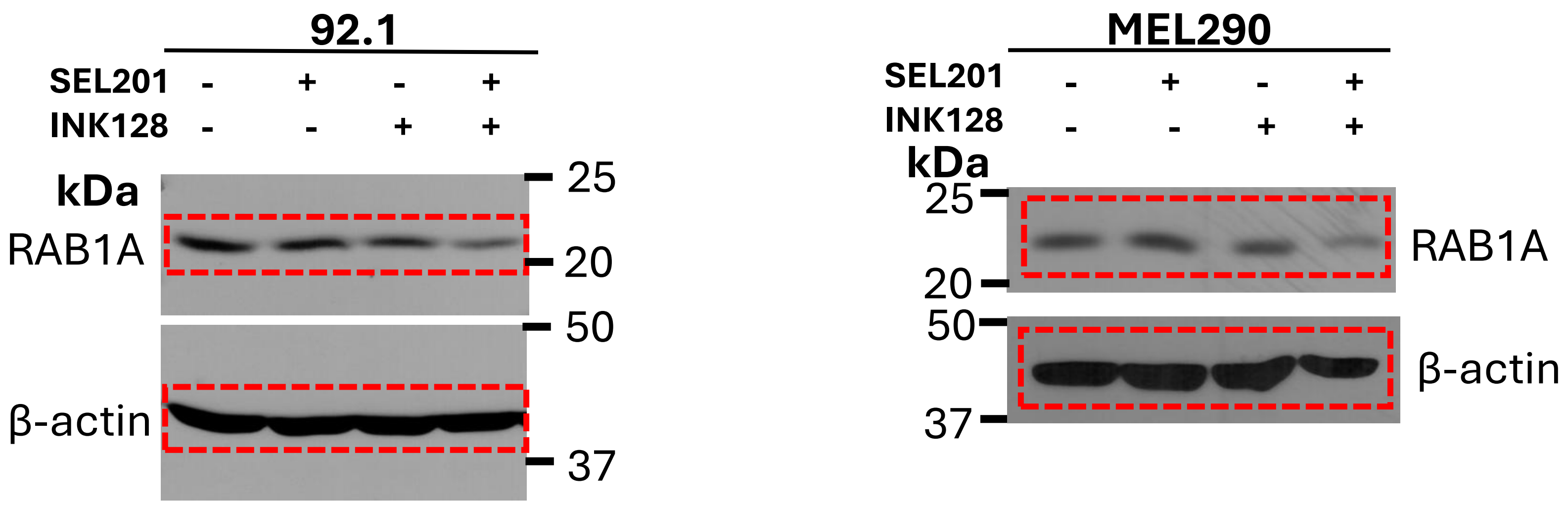

Figure 3A T128, T143, 92.1

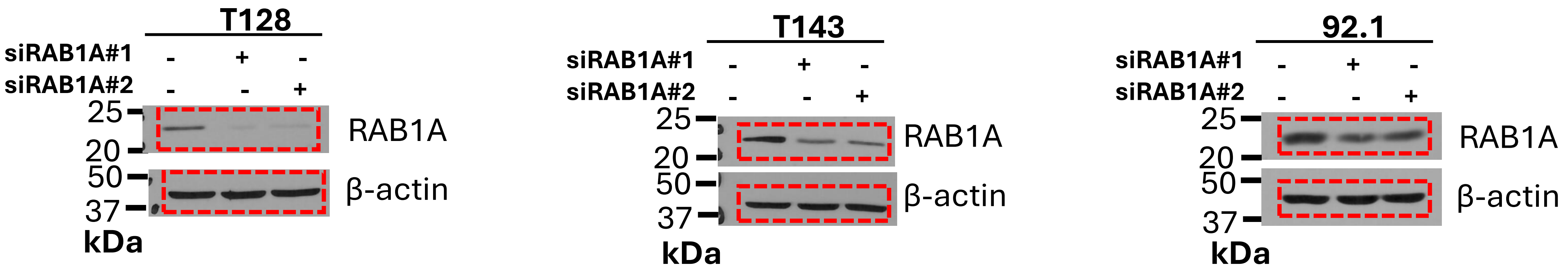

Figure 3C T128, T143

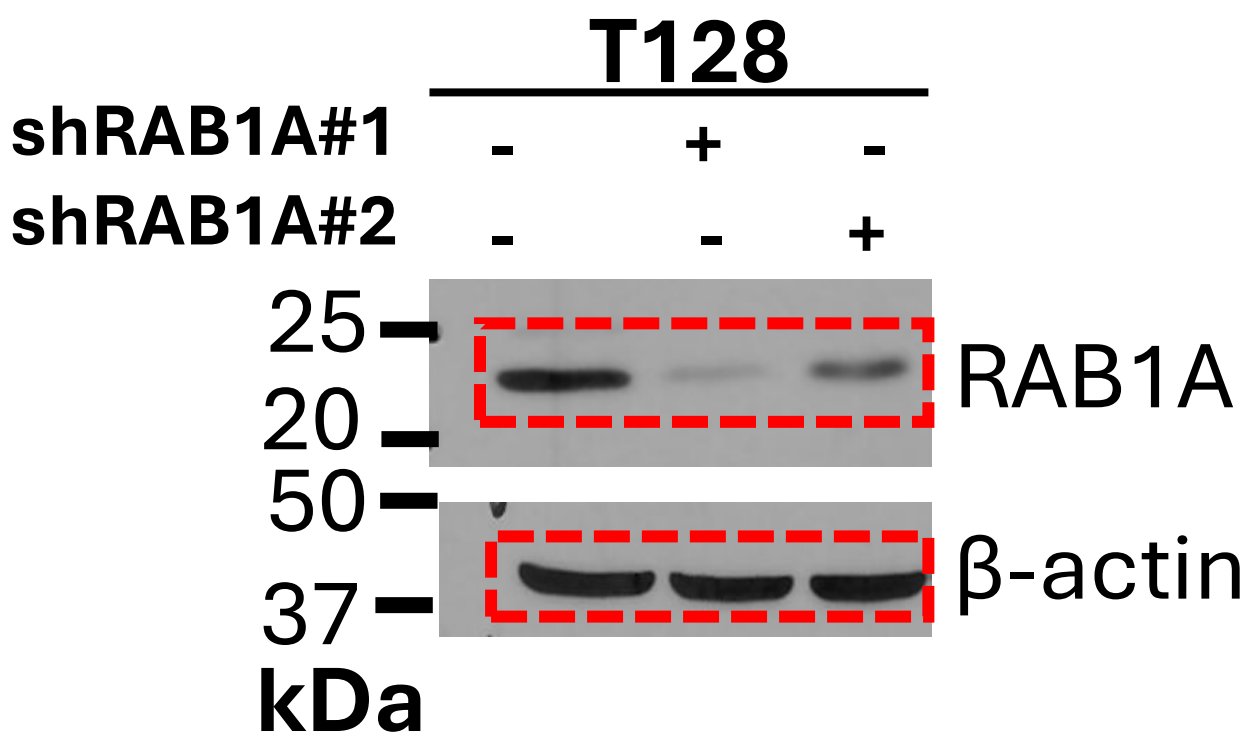

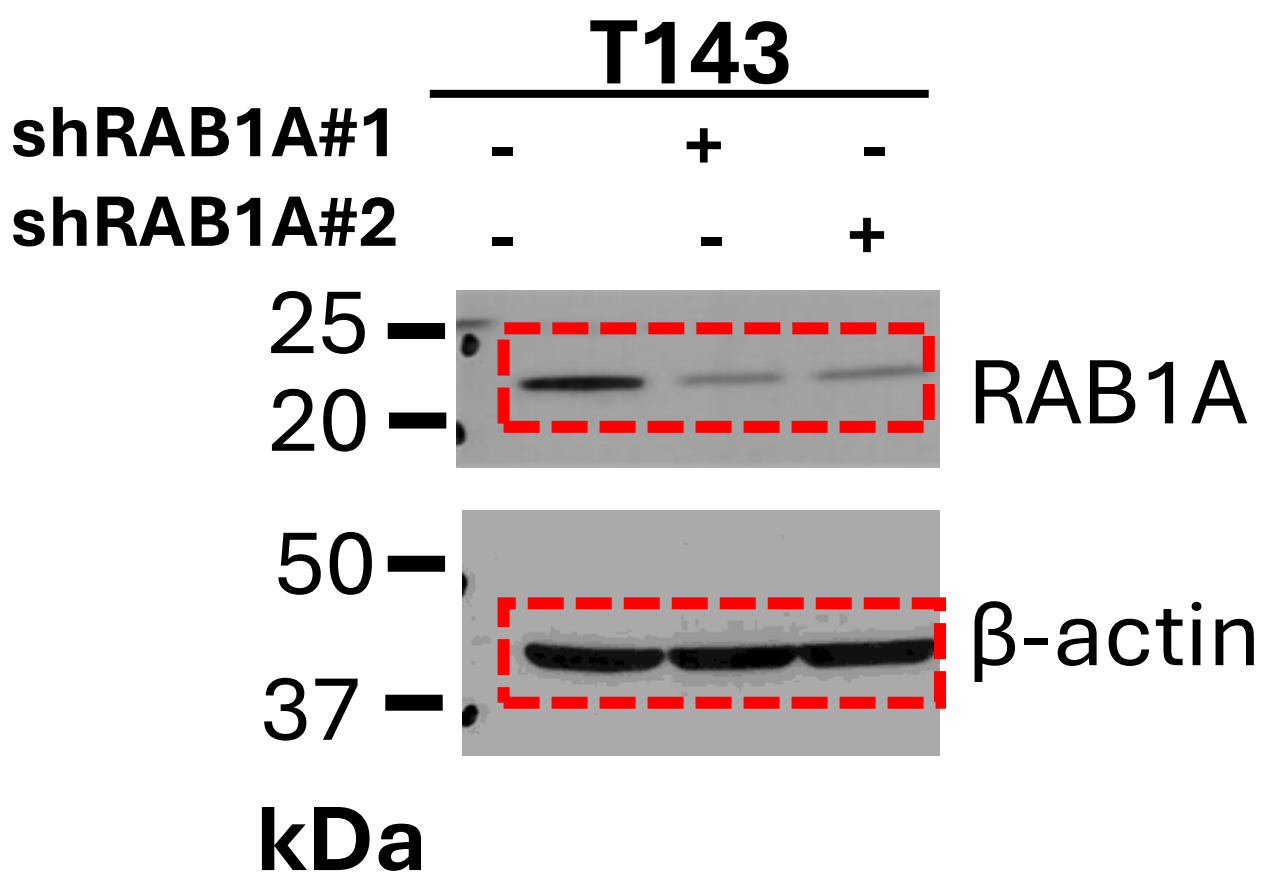

Figure 3D T128, T143

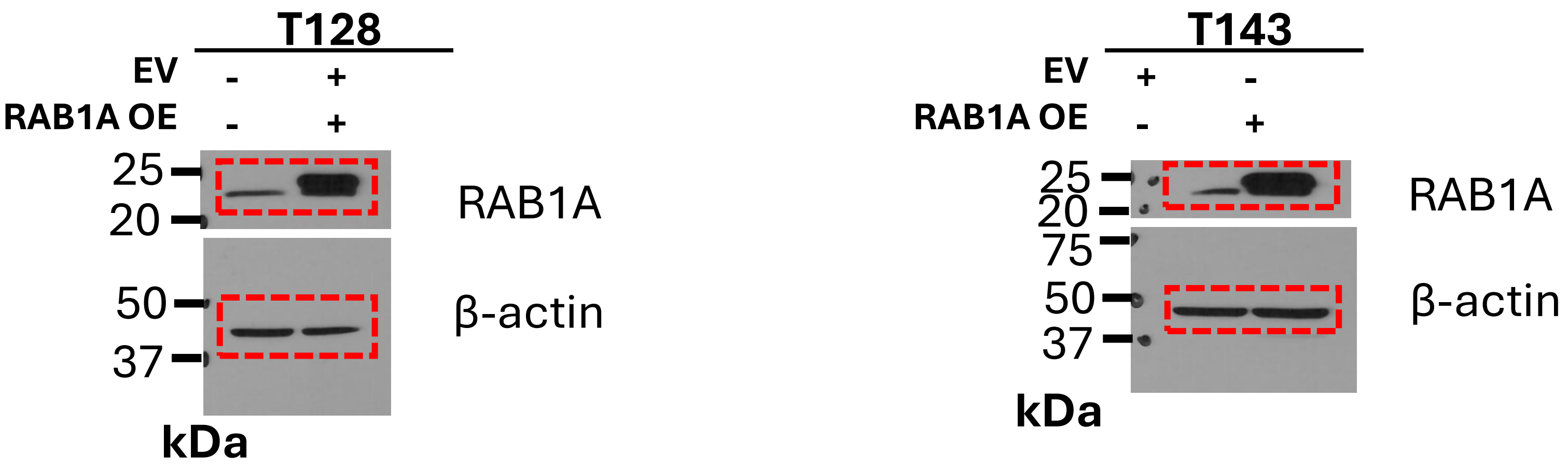

Figure 3I T128

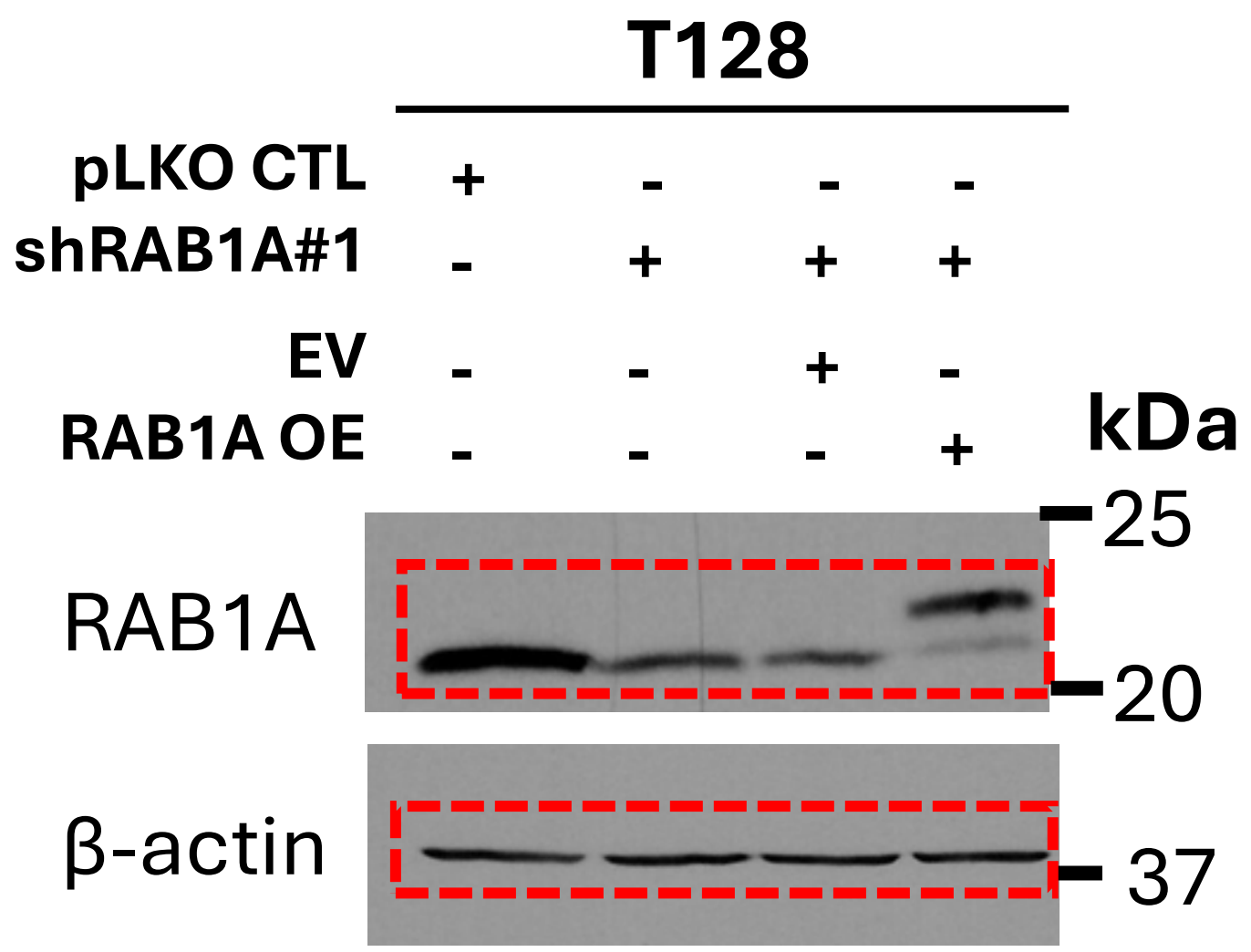

Figure S2A T143, T128

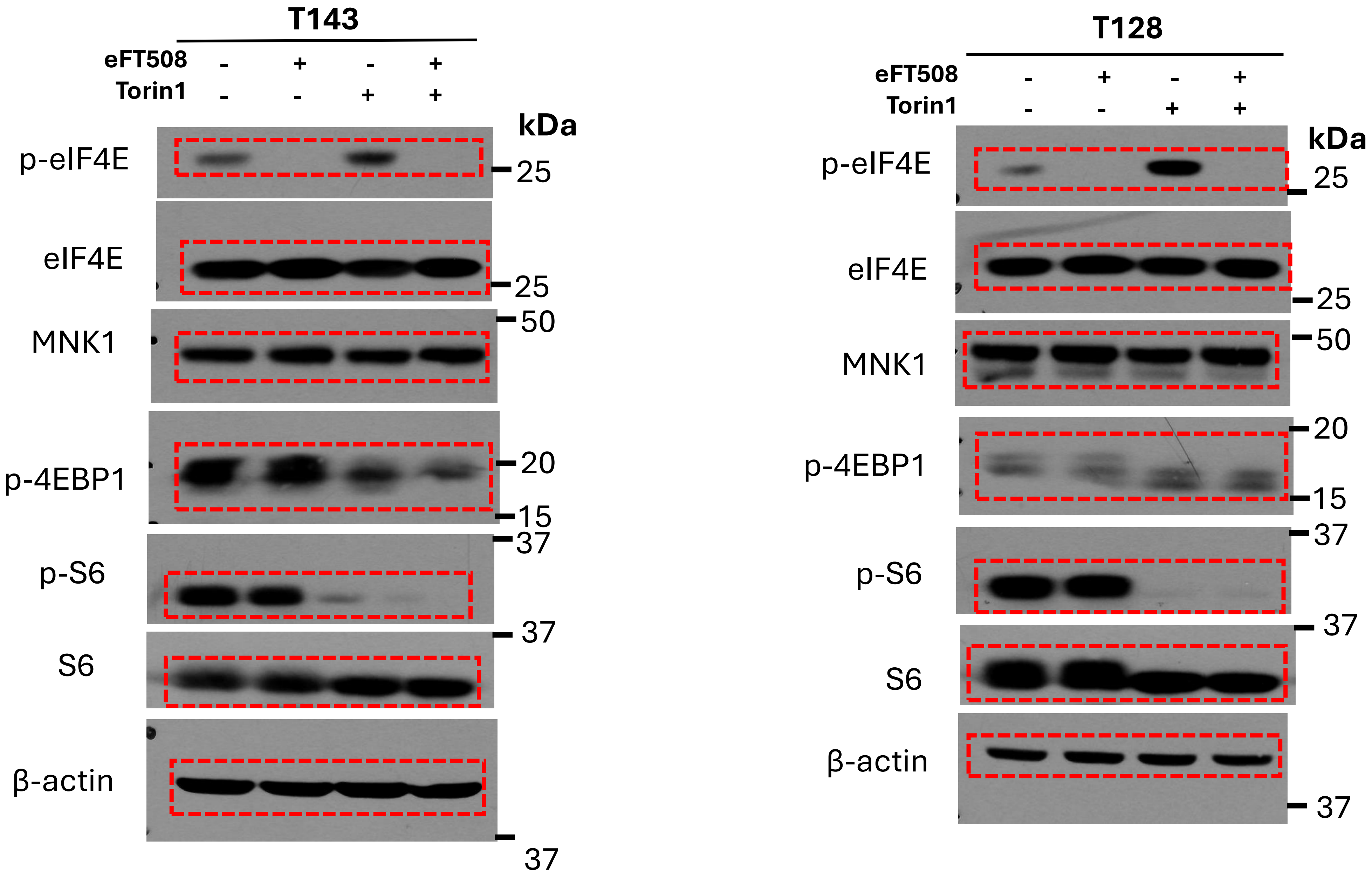

Figure S2A 92.1, MEL290

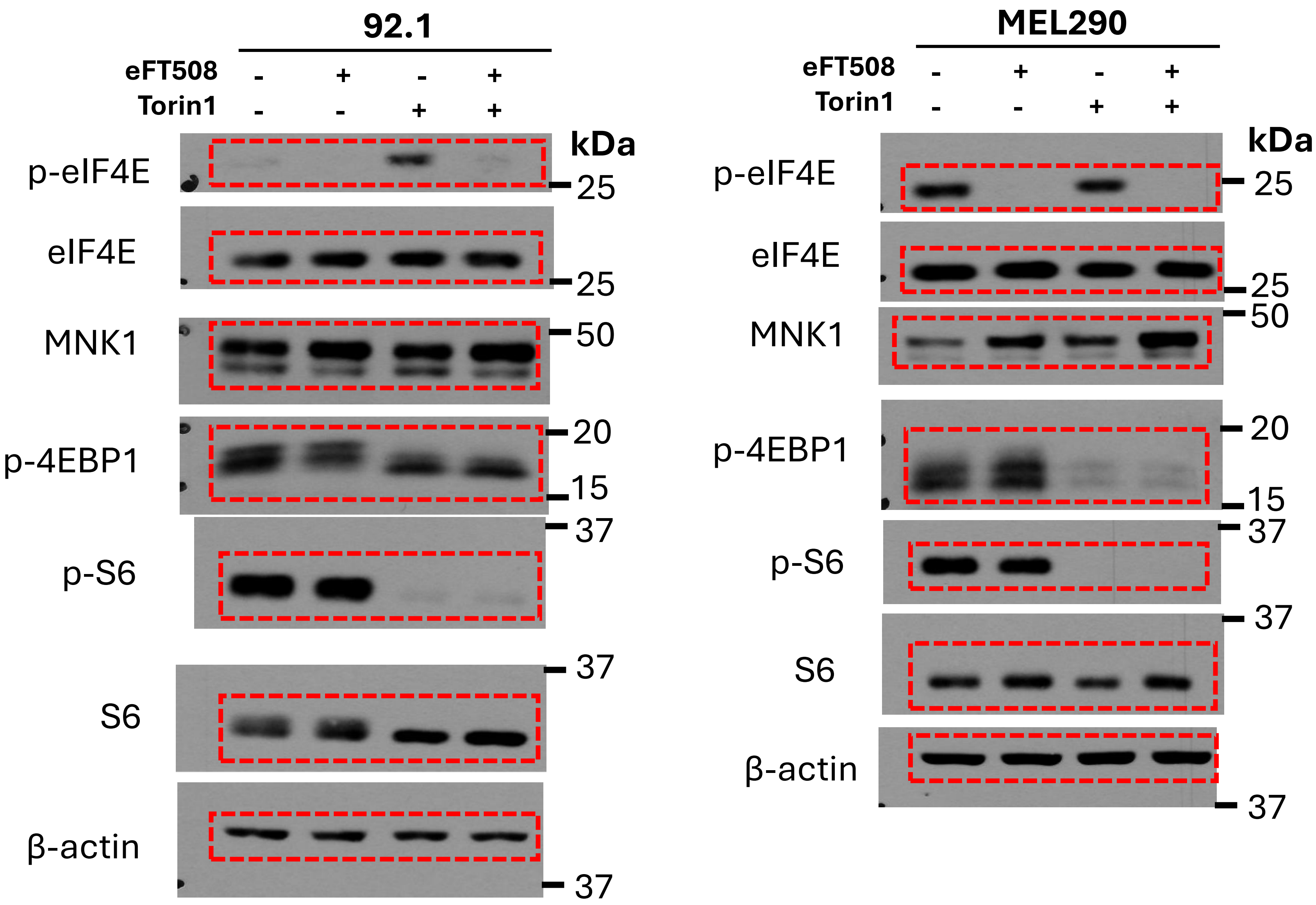

Figure S3A MEL270

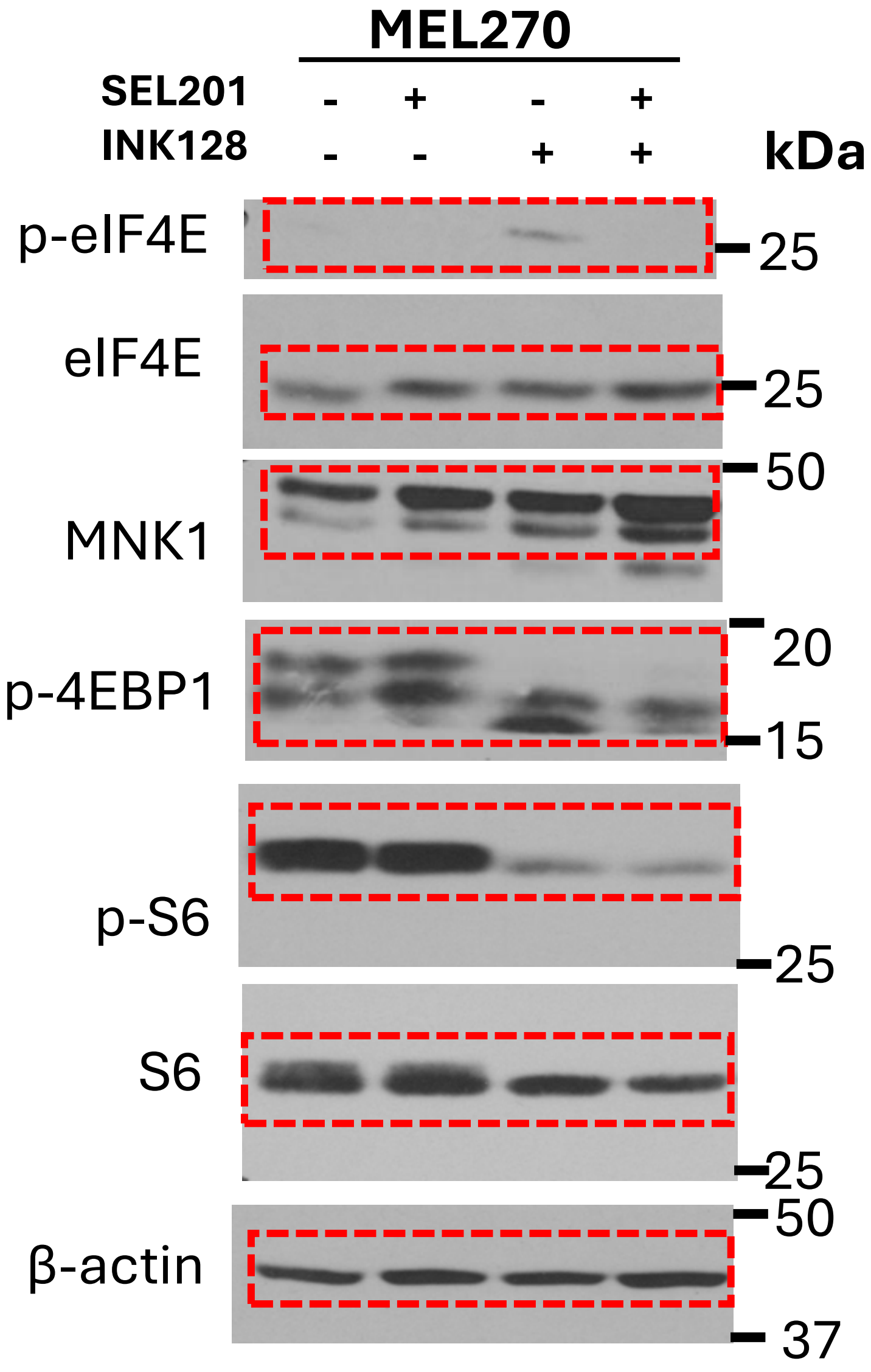

Figure S3A MP46

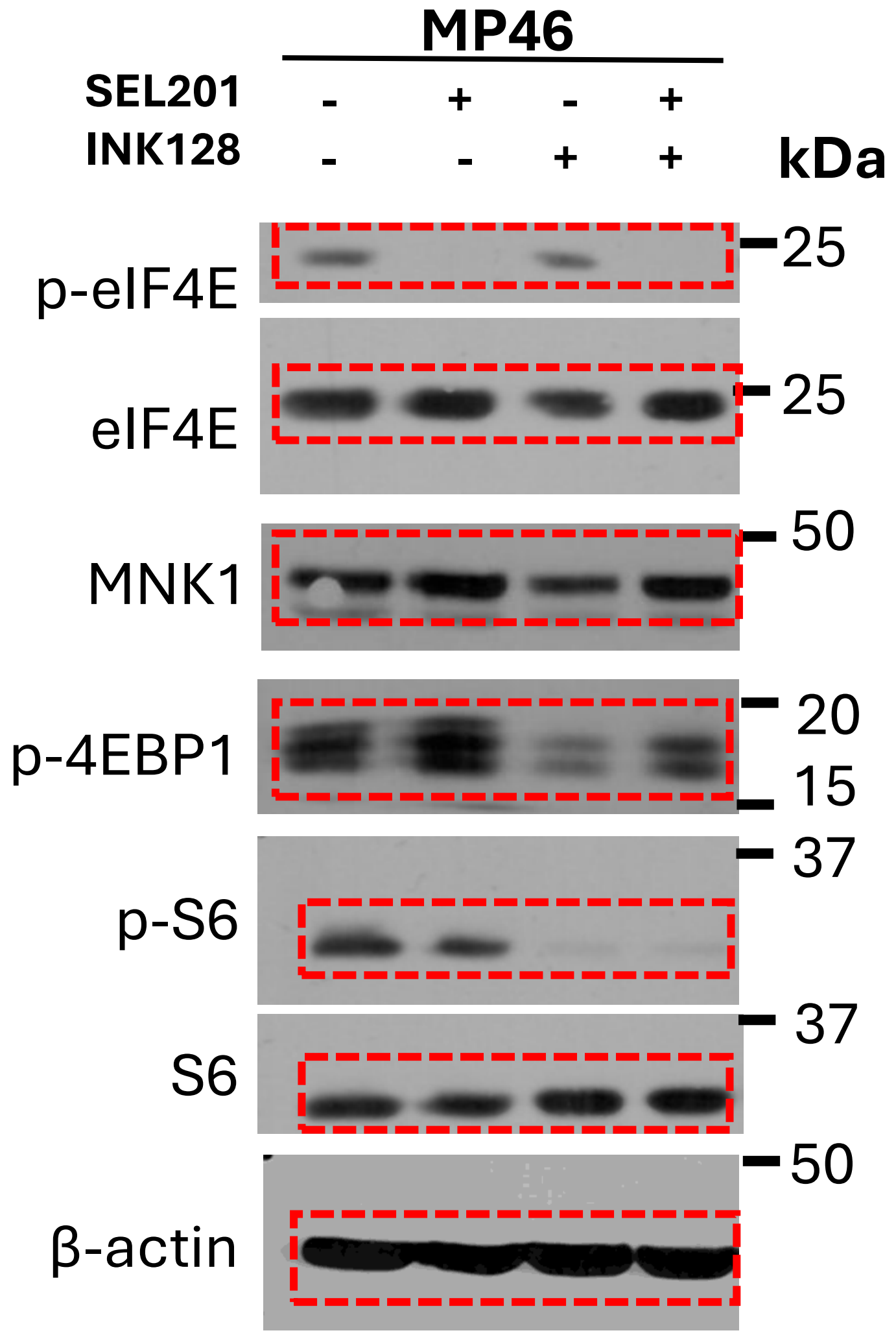

Supplement: Supplementary file 1 [file 41418_2026_1684_MOESM13_ESM.pdf]
