# Supplementary material for: RAB1A is a novel vulnerability in uveal melanoma revealed by dual inhibition of MNK1/2 and mTOR
Source: Cell Death Differ. 2026 Feb 18;33(9):1891–906. doi: 10.1038/s41418-026-01684-y (PMC13545286; doi:10.1038/s41418-026-01684-y)
Supplement: Supplementary file 2 [file 41418_2026_1684_MOESM10_ESM.docx]

SUPPLEMENTAL FIGURES

**Supplementary Fig. S1 MNK1/2-mTORC1/2 activation status in primary-derived human UM TMA.** **A** IHC stains of p-eIF4E, p-S6 and RAB1A on primary human UM TMA (n=28 cores). The cores were stained and graphed using pixel intensity and organized using patient ID, and later scored using 3 different levels of expression, corresponding to low (negative control stain), medium or high expressing. Exhausted cores shown with red underline were omitted from the analysis (n=3 cores).

**Supplementary Fig. S2 MNK1/2 and mTORC1/2 pharmacological inhibition, with eFT508 and Torin1 respectively, decreases UM clonogenic outgrowth and invasion.** **A** Immunoblot analysis of the indicated proteins in T128, T143, 92.1 and MEL290 UM cell lines using eFT508 (5µM) and Torin1 (25 nM) inhibitors, alone or in combination. **B, C** eFT508 cooperates with Torin1 to decrease clonogenicity and invasion in UM cell lines. Experiments represent the mean ±SD of 3 experimental replicates performed in technical triplicate. One-way ANOVA multiple comparisons test. ***P* ≤0.01, ****P* ≤0.001 and *****P* ≤0.0001 compared to single treatments.

**Supplementary Fig. S3 MNK1/2 and mTORC1/2 pharmacological inhibition decreases UM clonogenic and invasive outgrowth *in vitro*.** **A** Immunoblot showing the effects of SEL201 and INK128 on the indicated proteins in MEL270 and MP46 UM cell lines. **B** SEL201 cooperates with INK128 to decrease clonogenicity and invasion in MEL270 and MP46 UM cell lines. Experiments represent the mean ±SD of 3 experimental replicates performed in technical triplicate. One-way ANOVA multiple comparisons test. **P* ≤0.05,***P* ≤0.0, ****P* ≤0.001 and *****P* ≤0.0001 compared to single treatments.

**Supplementary Fig. S4** **Proteome profiling of T128 UM cell line shows that inhibition of MNK1/2 and mTORC1/2, alone or in combination, impacts multiple signaling pathways.** **A** Schematic of downstream analysis and hierarchical clustering of the differentially regulated proteins detected using DIA on T128 UM cells treated with vehicle, SEL201, INK128 or combination therapy at 24 hours. **B** Pathway enrichment analysis of cluster 1. **C** Pathways enrichment analysis of cluster 2. **D** Pathway enrichment analysis of cluster 5. **E** Pathway enrichment analysis of cluster 6. **F** Pathway enrichment analysis of cluster 7. Pathway enrichment analysis was not performed on cluster 3 due to small proteins number. **G** *RAB1A* mRNA expression in T128, T143, 92.1 and MEL270 treated with DMSO, SEL201, INK128 or SEL201+INK128, values were compared to *ACTB* as housekeeping gene. One-way ANOVA multiple comparisons test, ns=non-significant, **P* ≤0.05, ***P* ≤0.01, ****P* ≤0.001 and *****P* ≤0.0001.

**Supplementary Fig. S5** **Surfaceome analysis of differentially regulated proteins in shRAB1A and RAB1A OE vs controls.** **A, B** Volcano plots showing the differential protein expression at the plasma membrane of T128 cells transduced with shRNA against RAB1A compared to pLKO Control and RAB1A OE compared to empty vector (EV). Dashed lines depict significance (*P* < 0.001) and fold enrichment ≤ + and - 0.15 thresholds. **C, D** Pathway enrichment analysis and protein-protein interaction of surface markers downregulated in shRAB1A compared to pLKO Control. **E,** **F** Pathway enrichment analysis and protein-protein interaction of surface markers upregulated in RAB1A OE compared to EV.

**Supplementary Fig. S6 Survival analysis of differentially regulated proteins in shRAB1A and RAB1A OE vs controls in TCGA UM dataset. A-D** Kaplan-Meier survival analysis showing RAB1A, CADM1, LRP8, and EPHB2 expression in the TCGA UVM dataset was analyzed by subgrouping using tumor stage III and IV (n=34).

**Supplementary Fig. S7 Flow cytometry to detect CADM1 at the cell membrane. A-D** Percentage of cells expressing CADM1 in T128 cells transfected with shRAB1A, RAB1A OE or shCADM1, RAB1A OE+shCADM1 when compared to their respective controls. **E**, CADM1 expression is reduced via shRNA at the cell membrane in RAB1A OE T128 cells, when compared to its control. **F-J,** Flow gating strategy and dot plots for the data shown in A-E and Fig. 7I-K.

**Supplementary Fig. S8 Weight of mice treated with DMSO, SEL201, INK128 or combination treatment. A** Bi-weekly weight monitoring of NCG bearing T128 luc-tdTomato tumors treated with vehicle (n=6), SEL201 (n=5), INK128 (n=5) and combination treatments (n=7), two-way ANOVA multiple comparisons test, ns=non-significant at endpoint (day 17).

**Supplementary Fig. S9**. **A-F** Representative images corresponding to the colony formation and invasion assays described in the main figures**.** Labels at the top of each panel indicate the assay shown.
