# Supplementary material for: RAB1A is a novel vulnerability in uveal melanoma revealed by dual inhibition of MNK1/2 and mTOR
Source: Cell Death Differ. 2026 Feb 18;33(9):1891–906. doi: 10.1038/s41418-026-01684-y (PMC13545286; doi:10.1038/s41418-026-01684-y)
Supplement: Supplementary file 3 [file 41418_2026_1684_MOESM11_ESM.docx]

SUPPLEMENTAL TABLES

Supplementary Table S1. Information of primary antibodies used for immunoblotting, immunohistochemistry, immunofluorescence, and primers used for Quantitative PCR assays.

| **Target** | **Antibody Source/Catalog (#) or sequence** | **Application** | **Dilution** |
| --- | --- | --- | --- |
| p-eIF4E | Cell Signaling #9741 | Immunoblot | 1:1000 |
| eIF4E | BD Biosciences #610270 | Immunoblot | 1:1000 |
| MNK1 | Cell Signaling #2195 | Immunoblot | 1:1000 |
| p-4EBP1 | Cell Signaling #2855 | Immunoblot | 1:1000 |
| 4EBP1 | Cell Signaling #9452 | Immunoblot | 1:1000 |
| p-S6 | Cell Signaling #5364 | Immunoblot | 1:1000 |
| S6 | Cell Signaling #2317 | Immunoblot | 1:1000 |
| β-Actin | Sigma Aldrich #A5441 | Immunoblot | 1:5000 |
| RAB1A | Proteintech #11671-1-AP | Immunoblot | 1:1000 |
| p-eIF4E | Abcam #ab76256 | Immunohistochemistry | 1:50 |
| p-S6 | Cell Signaling #35708 | Immunohistochemistry | 1:200 |
| RAB1A | Abcam # ab302545 | Immunohistochemistry | 1:100 |
| Nucleolin | Abcam # ab136649 | Immunohistochemistry | 1:2000 |
| RFP | Rockland #600-401-379 | Immunohistochemistry | 1:300 |
| HA-Tag | Cell Signaling #3724 | Immunofluorescence | 1:800 |
| Anti-GFP | Santa Cruz #sc9996 | Immunofluorescence | 1:1000 |
| Anti-GM130 | Cell Signaling #12480 | Immunofluorescence | 1:3000 |
| CADM1 | Abcam # ab216585 | Flow cytometry | 1:100 |
| AF647-conjugated anti-rabbit | ThermoFisher Scientific #A-31573 | Flow cytometry | 1:400 |
| *RAB1A* FW | GGGAAACCAATCAAGCTTCAAA | Quantitative PCR | 1 µM |
| *RAB1A* RV | CTGGAGGTGATTGTTCGGAAT | Quantitative PCR | 1 µM |
| *ACTB* FW | AGGCACCAGGGCGTGAT | Quantitative PCR | 1 µM |
| *ACTB* RV | GCCCACATAGGAATCCTTCTGAC | Quantitative PCR | 1 µM |
